# Low-Density Polybutylene Terephthalate Foams with Enhanced Compressive Strength via a Reactive-Extrusion Process

**DOI:** 10.3390/polym12092021

**Published:** 2020-09-04

**Authors:** Merve Aksit, Sebastian Gröschel, Ute Kuhn, Alper Aksit, Klaus Kreger, Hans-Werner Schmidt, Volker Altstädt

**Affiliations:** 1 Department of Polymer Engineering, University of Bayreuth, Universitaetsstrasse 30, 95447 Bayreuth, Germany; merve.aksit@uni-bayreuth.de (M.A.); sebastian.groeschel@uni-bayreuth.de (S.G.); ute.kuhn@uni-bayreuth.de (U.K.); alper.aksit@uni-bayreuth.de (A.A.); 2 Macromolecular Chemistry 1, University of Bayreuth, Universitaetsstrasse 30, 95447 Bayreuth, Germany; klaus.kreger@uni-bayreuth.de; 3 Bavarian Polymer Institute and Bayreuth Institute of Macromolecular Research, University of Bayreuth, Universitaetsstrasse 30, 95447 Bayreuth, Germany

**Keywords:** foams, polybutylene terephthalate, foam extrusion, reactive-extrusion, supramolecular additives, 1,3,5-benzene-trisamides, cell nucleation, foam morphology, compressive strength

## Abstract

Due to their appealing properties such as high-temperature dimensional stability, chemical resistance, compressive strength and recyclability, new-generation foams based on engineering thermoplastics such as polyethylene terephthalate (PET) and polybutylene terephthalate (PBT) have been gaining significant attention. Achieving low-density foams without sacrificing the mechanical properties is of vital importance for applications in the field of transportation and construction, where sufficient compressive strength is desired. In contrast to numerous research studies on PET foams, only a limited number of studies on PBT foams and in particular, on extruded PBT foams are known. Here we present a novel route to extruded PBT foams with densities as low as 80 kg/m^3^ and simultaneously with improved compressive properties manufactured by a tandem reactive-extrusion process. Improved rheological properties and therefore process stability were achieved using two selected 1,3,5-benzene-trisamides (BTA1 and BTA2), which are able to form supramolecular nanofibers in the PBT melt upon cooling. With only 0.08 wt % of BTA1 and 0.02 wt % of BTA2 the normalized compressive strength was increased by 28% and 15%, respectively. This improvement is assigned to the intrinsic reinforcing effect of BTA fibers in the cell walls and struts.

## 1. Introduction

Extruded foams of semicrystalline polymers combine attractive properties such as high-temperature dimensional stability, chemical resistance, strength and stiffness, as well as recyclability. These benefits make semicrystalline foams based on commodities and engineering plastics such as isotactic polypropylene (i-PP), PET and PBT promising candidates for a broad diversity of applications. For instance, these foams are applied as packaging and insulation materials, in the field of rail and road transportation, wind energy, marine as well as building and construction [1,2,3]. For all these applications, an ongoing task is to reduce the material weight without significant deterioration of the mechanical properties [2].

In foam extrusion, however, the poor rheological properties of semicrystalline polymers at the typical high processing temperatures limit their use. For instance, at very high temperatures (upper-temperature limit), sagging and cell collapse occur due to very low melt viscosity and strength when the polymer melt encounters uniaxial and biaxial deformations (cell nucleation and growth). At lower temperatures (lower-temperature limit), polymer crystallization is initiated rapidly. As a result of the polymer solidification, the maximum allowable barrel pressure in the extruder is exceeded, causing a shut-down of the machine [4]. The combination of the low values of melt viscosity, melt strength and elasticity, as well as the quick solidification causes a narrow temperature-processing window and restricts the versatility and flexibility of the foaming process. Both cases give foams having high densities and inhomogeneous and coarse cell morphology [5]. Therefore, improving the rheological properties of these polymers is crucial [6]. To address this issue, several strategies have been developed to improve the melt viscosity and melt strength as well as the strain hardening (extensional thickening) behavior, which is mainly caused by increased molecular weight and intrinsic entanglements in the polymer melt. Additional entanglements as well as an increase in molecular weight can be introduced by post-polymerization modification reactions such as partial crosslinking [7], long-chain branching and chain extension [6,8,9,10,11,12]. Chain extension or branching has been mostly used for the semicrystalline polyesters, PET and PBT [6,13,14]. As reagents for the post-polymerization modification reactions, cyclic anhydrides [15,16], oxazolines [12], carbodiimides [17], isocyanates and epoxides [18,19] are often used, which react with the hydroxyl and carboxyl end groups of the polyester. Chemically post-modified PET offers higher zero shear viscosity and higher extensional viscosity widening the processing window. This provides foams with fine and homogeneous cell morphology and improved properties [14].

In contrast to numerous studies on the modification and foaming of PET [7,8,10,16,18,20], a limited number of studies on the modification of PBT via reactive extrusion [21,22,23,24] and very few studies on extrusion foaming of PBT [2,14,25] are known. Yet, PBT is a promising candidate to be used in the polymer foam market, because it features several advantages. For instance, due to its flexible and mobile butylene groups, PBT can crystallize fast and efficiently so that it generates a well-ordered molecular structure with appealing mechanical properties of the resin. Furthermore, its easy-processability and lower melting temperature require less processing energy resulting in less of an environmental impact [26]. Moreover, PBT exhibit also high-temperature dimensional stability, high heat deflection temperature (up to 150 °C), chemical and abrasion resistance as well as high toughness and good electrical properties increase. These properties render PBT foams suitable for diverse applications such as insulation or sound absorption in the engine compartment, as a core material for sandwich structures in the aircraft and automotive industry or for packaging, construction and transportation applications [2,14,20,25,27].

The first example of PBT foams was reported in 1997 by Klotzer et al. [28]. They achieved extruded PBT foams with a coarse cell morphology using a physical blowing agent (PBA). Song et al. produced PBT foams by using a technology called “Dynamic Decompression and Cooling (DDC) Process” [27]. With this method, they obtained PBT foams with an open-cell structure and structural anisotropy. Jeong et al. produced PBT foams with the chemical blowing agent, 5-phenyltetrazole by means of a conventional lab-scale foam extruder (length to diameter ratio; L/D = 20, from Brabender) [25]. They reported a minimum foam density and cell size of 600 kg/m^3^ and 500 µm, respectively. In a further study, they modified PBT by using triglycidyl isocyanurate as a chain extender (CE) to improve the rheological properties [14]. After dry blending of PBT with the CE, PBT foams were manufactured via reactive extrusion (single screw extruder, D = 32 mm, L/D = 40, Killion) in the presence of isobutene as PBA. This led to a decrease in foam density from 510 to 330 kg/m^3^. However, the cell size increases from 91 to 140 μm and thus the cell density decreases from 1.48 × 10^6^ to 3.56 × 10^5^ cells/cm^3^.

In this context, we have demonstrated the applicability of foam injection molding of PBT with a breathing mold. By using nitrogen as a physical blowing agent, a maximum density reduction of the PBT foam was found to be around 50%, which features lower densities compared to PBT foams processed with chemical blowing agents [29]. We have also successfully applied bead foaming processes to achieve PBT foams with low-to-medium densities (<500 kg/m^3^). By means of a tandem extrusion line (twin screw extruder, D = 25 mm, L/D = 42; single-screw extruder, D = 45 mm, L/D = 30, Dr. Collin GmbH) with 3 wt % supercritical carbon dioxide (s-CO_2_) as a PBA in combination with an underwater granulation unit, expanded-PBT (e-PBT) bead foams were produced. The average density of e-PBT bead foams was found to be 230 kg/m^3^. By improving the rheological properties of the PBT, the density of the bead foams could be further reduced to around 160 kg/m^3^ [6,30]. This improvement of the rheological properties was achieved by (I) introducing long-chain branching with 1 wt % of a multifunctional epoxy-based CE and (II) optimizing the processing conditions including the foaming and die temperature, the PBA concentration as well as the water temperature and the blade speed of the underwater granulate unit. Remarkably, the tensile modulus of e-PBT samples was similar and compressive strength at elevated temperatures was higher compared to a reference foam, expanded-polypropylene.

In general, insoluble solid additives for semicrystalline polymers are widely used, acting for example as crystal nucleating agents, foam nucleating agents, fillers and reinforcement additives. The mostly used insoluble additives for semicrystalline polymer foams are talc [31], clay [32], graphite [33], carbon nanofibers [34], calcium carbonate [35], hollow glass microspheres [36], sodium benzoate [37], zeolite [37], polytetrafluoroethylene [38] and in-situ fibrillated polymer fibers such as PET [39] and PP [40]. Besides these insoluble additives, supramolecular polymer additives were also applied. From the plethora of the supramolecular building blocks, a prominent example is based on the material class of 1,3,5-benzene-trisamides (BTA). Depending on the polymer type, additive concentration and conditions such as the processing temperature, BTAs can be dissolved at a molecular level in the polymer melt. Upon cooling, they self-assemble into columnar stacks forming supramolecular nano-objects. These nanostructures are able to function as polymer crystal nucleating agents, foam nucleating agents and electret additives for various types of polymers such as polystyrene (PS) i-PP and polyvinylidene fluoride [3,41,42,43,44,45,46,47,48,49]. Apart from these non-polar polymers, identifying suitable supramolecular polymer additives, which can also act as nucleation agents for the polymer crystallization of PBT, is more challenging due to the more polar character and higher processing temperatures. Thus, we have comprehensively studied the solubility and self-assembly behavior of BTAs in the PBT melt as well as their suitability to provide an epitaxial surface inducing the polymer crystallization [50]. We found that for selected BTAs the nano-objects are formed in-situ in the polymer melt at elevated temperatures. Moreover, the resulting nano-object size and nucleation efficiency strongly depend on the individual chemical structure of BTA, whereby some of the BTAs provided a significant increase in the crystallization temperature of PBT, while other BTAs are present as nano-objects but could not nucleate PBT [50].

Herein, we select two different 1,3,5-benzene-trisamides (BTAs), which are known to form nanofibers upon cooling in the PBT polymer melt at different concentrations, allowing us to study their influence on the extensional viscosity and on the foaming process of PBT. Using a chain extender to modify the rheological properties of PBT, we establish structure–property relationships of the extruded PBT foams featuring different concentrations of the BTAs with respect to the foam morphology and the compressive strength. Neat PBT, which is modified with 0.6 wt % CE, was used as a reference material because non-modified neat PBT exhibits poor melt strength and foamability, resulting in improper foam morphology with partially non-foamed areas.

## 2. Materials and Methods

### 2.1. Materials

**Figure 1:**
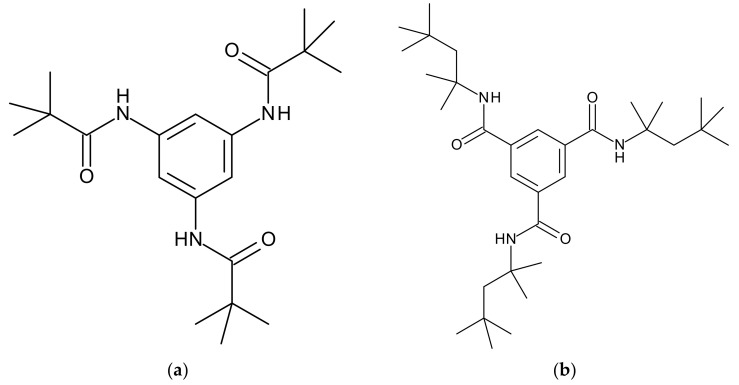
Chemical structures of (**a**) BTA1 and (**b**) BTA2.

Two supramolecular polymer additives based on 1,3,5-benzene-trisamides were selected for this study, because both BTA1 (*N*,*N*’,*N*’’-1,3,5-benzenetriyltris(2,2-dimethylpropanamide)) and BTA2 (*N*,*N*’,*N*’’-tert-octyl-1,3,5-benzene-tricarboxylic acid) are known to form nanofibers in the PBT polymer melt but feature a different solubility behavior. Their chemical structures are shown in Figure 1a,b, respectively. BTA1 (Irgaclear^®^ XT 386) is commercially available from BASF SE (Ludwigshafen, Germany) [51], and BTA2 was synthesized and characterized according to literature procedures [52,53,54].

PBT (Ultradur^®^ B 6550) was received from BASF SE (Ludwigshafen, Germany). The PBT has a weight average molecular weight (M_w_) of 90,000 g/mol and a number average molecular weight (M_n_) of 34,000 g/mol was determined, yielding a polydispersity index of 2.65. It features a density of 1300 kg/m^3^ and a melt volume rate (MVR) of 9.5 cm^3^/10 min at 250 °C and 2.16 kg [55]. Prior to processing, PBT was dried overnight at 120 °C under vacuum.

**Figure 2:**
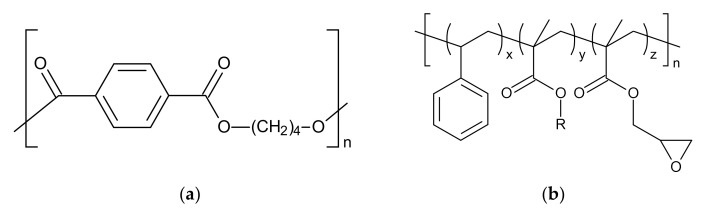
Chemical structures of (**a**) PBT and (**b**) Joncryl^®^ ADR 4468.

For the post-modification of the PBT, an epoxy-based CE (Joncryl^®^ ADR 4468) from BASF SE (Ludwigshafen, Germany) was purchased. This CE is a multifunctional reactive copolymer with an epoxy equivalent weight of 315 g/mol and a M_w_ of 6978 g/mol [56]. The chemical structures of PBT and CE are shown in Figure 2a,b.

**Figure A1:**
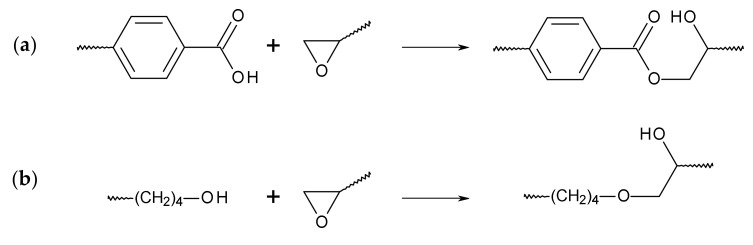
General scheme of two types of post modification reactions between the terminal end groups of PBT and an epoxy-based chain extender (CE): (**a**) esterification and (**b**) etherification.

**Figure A2:**
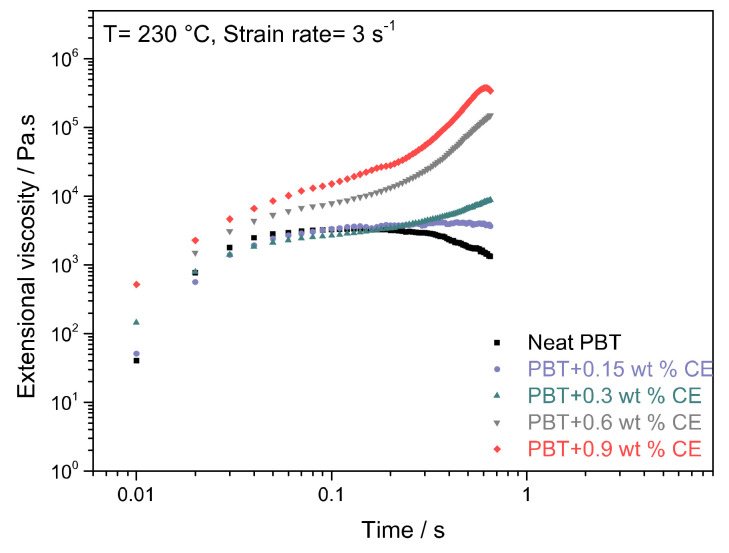
Extensional viscosities of the neat PBT and PBT modified with 0.15, 0.3, 0.6 and 0.9 wt % of the epoxy-based CE.

**Figure A3:**
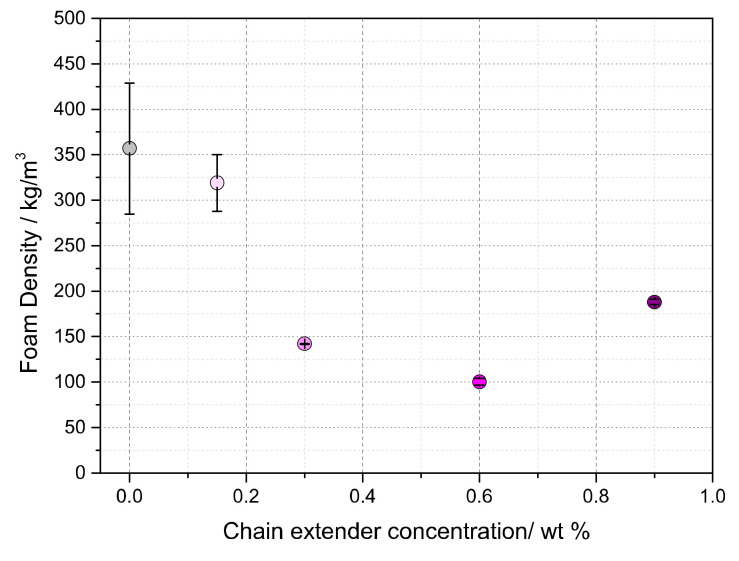
Foam densities of the neat PBT and PBT modified with 0.15, 0.3, 0.6 and 0.9 wt % of the epoxy-based CE.

**Figure A4:**
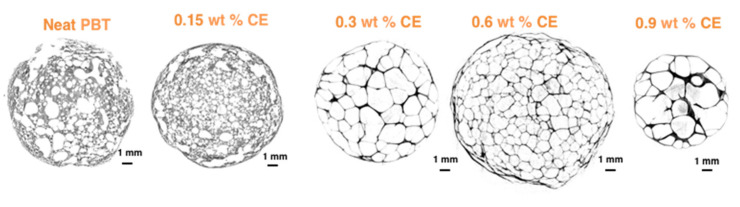
µ-CT images of the neat PBT foam and PBT foams modified with 0.15, 0.3, 0.6 and 0.9 wt % of the epoxy-based CE.

Two possible post-modification reactions of PBT with an epoxy-based CE are known, as illustrated in Figure A1 [14]. These include either the esterification of the carboxyl terminal groups of the polyester or the etherification of the hydroxyl terminal groups of the polyester with the epoxy moieties of the CE. Four different concentrations (0.15, 0.3, 0.6 and 0.9 wt %) of the CE were mixed with PBT, compounded and evaluated. PBT with 0.6 wt % of the CE was identified as the optimum concentration in terms of extensional melt viscosity (Figure A2) and foamability of PBT as well as achieving minimum foam density (Figure A3) with uniform morphology (Figure A4). 0.15 wt % and 0.3 wt % of the CE were not sufficient to provide a proper strain hardening. In contrast, a concentration of 0.9 wt % deteriorates the process stability, resulting in non-uniform PBT foams with higher foam densities. In the following, only modified PBT with 0.6 wt % of CE is used, which is denoted as mPBT.

### 2.2. Reactive Foam Extrusion Process

Foams based on modified PBT with 0.6 wt % of CE (mPBT) and based on mPBT with concentrations of 0.02, 0.04, 0.08, 0.1 and 0.25 wt % of BTA1 and BTA2 were processed using a tandem extrusion line from Dr. Collin GmbH (Ebersberg, Germany; twin-screw extruder (A-Extruder) with 25 mm screw diameter and L/D = 42; single-screw extruder (B-Extruder) with 45 mm screw diameter and L/D = 30) equipped with a round-die with a diameter of 3 mm. As a physical blowing agent, 2 wt % s-CO_2_ was used. For the processing of each foam compound, powder mixtures comprising of the respective amount of PBT, CE and BTA (masterbatch) were prepared. Hopping of masterbatches were done by a powder hopper while PBT was fed by a granulate hopper to the A-extruder. Uniform dissolution of BTAs and s-CO_2_ in the PBT melt as well as the post-modification of PBT with the CE took place in the A-extruder under pressure. The PBT melt was then cooled down in the B-extruder to a respective temperature, whereby the foaming was initiated at the die. The temperature of the melt at the exit of the B-extruder prior to the die is recorded for each sample. Neat mPBT was produced in the same manner as the reference material. The schematic representation of the tandem-extrusion line has been already presented and described in our previous studies [3,46]. The relevant processing parameters to produce neat mPBT foam and mPBT foams with different BTA concentrations for physical, thermal, morphological and mechanical characterizations are shown in Table 1.

### 2.3. Rheological Analysis

For the rheological analysis, compact granulates of the neat mPBT and mPBT with BTA1 and BTA2 were produced in the same manner by using the tandem extrusion line from Dr. Collin GmbH having the identical temperature profile and throughput of 5 kg/h. The granulates were then compression molded (P/O/Weber, Remshalden, Germany) to rectangular samples with the geometry of 18 mm × 10 mm × 0.8 mm to be used in extensional viscosity fixture measurements. The extensional viscosities were determined with the rheometer RDA III (Rheometrics Scientific, Piscataway, NJ, USA) equipped with a universal extension tool, at 230 °C and a Hencky strain rate of 1 s^−1^ as extensional strain rates during the foaming process appear around 1 s^−1^ [57,58]. For determination of the strain hardening coefficient (S), start-up shear experiments for each sample at a strain rate of 0.001 s^−1^ and temperature of 230 °C were conducted by using an Anton Paar MCR702 (Graz, Austria). The coefficient S is also described as a deviation from the Trouton relation ratio towards higher extensional viscosities. The Trouton ratio can be defined as a relation where the transient extensional viscosity equals three times the transient shear viscosity in the linear viscoelastic regime, which can be found by start-up shear experiments. Equation (1) shows the calculation of time-dependent coefficient $S\left(t,\dot{\varepsilon }\right)$ [59]:

(1) $$S\left(t,\dot{\varepsilon }\right):=\frac{\eta_{E}^{+}\;\left(t,\dot{\varepsilon }\right)}{3\eta^{+}\left(t\right)}$$

where $\eta_{E}^{+}\;\left(t,\dot{\varepsilon }\right)$ is the transient extensional viscosity as a function of time (t) and Hencky strain ($\dot{\varepsilon }$), and $\eta^{+}\left(t\right)$ is the transient shear viscosity in the linear viscoelastic region. The S values were calculated at t = 2 s, which is quite relevant for the foam extrusion process as the foam expansion time takes approximately 2 s [5].

### 2.4. Foam Characterization

#### 2.4.1. Foam Density

The AG245 analytical balance with a density-kit from Mettler Toledo (Columbus, OH, USA) was used for density measurements of foam samples according to ISO 1183-1 by applying the Archimedes’ principle. At least five samples from various locations of the foam strand were selected to be measured.

#### 2.4.2. Foam Morphology

The morphology of foam samples was visualized by microcomputer tomography (µ-CT) Skyscan 1072 100 kV from Bruker (Kontich, Belgium) with an acceleration voltage of 71 kV and a tubular flow of 142 μA. Foam cell areas A (μm^2^) were determined by analyzing reconstructed X-ray projections using the software ImageJ (University of Wisconsin, Madison, WI, USA). With the assumption of cells being circular, the cell diameter, D was calculated with Equation (2) [46]:

(2) $$D=2\sqrt{A/\pi }$$

To determine the cell densities ρ_c_ (cells/cm^3^), Equation (3) was used:

(3) $$\rho_{c}={\left(\frac{N_{c}}{A_{s}}\right)}^{\frac{3}{2}}$$

where N_c_ is the number of cells in the selected area and A_s_ (cm^3^) is the area of the selected section. Data analysis on the cell size and density were determined based on a total number of cells per cross-section of the foam strand.

Furthermore, a field emission-scanning electron microscopy (FE-SEM) Zeiss LEO 1530 from Carl Zeiss AG (Jena, Germany) was used with an acceleration voltage of 3 kV to visualize the BTA fibers. To do so, the surface of the PBT foam samples was hydrolyzed in a slowly stirred solution of 10 wt % sodium hydroxide (NaOH) in distilled water (H_2_O) for 2.5 h at 110 °C. Afterwards, the samples were carefully washed with water to remove residual NaOH and left overnight for drying at room temperature and atmospheric pressure. Prior to FE-SEM, the samples were coated with platinum having a layer thickness of 0.8 nm by using high resolution sputter coater Cressington 208HR (Watford, UK).

#### 2.4.3. Compressive Properties

**Figure A5:**
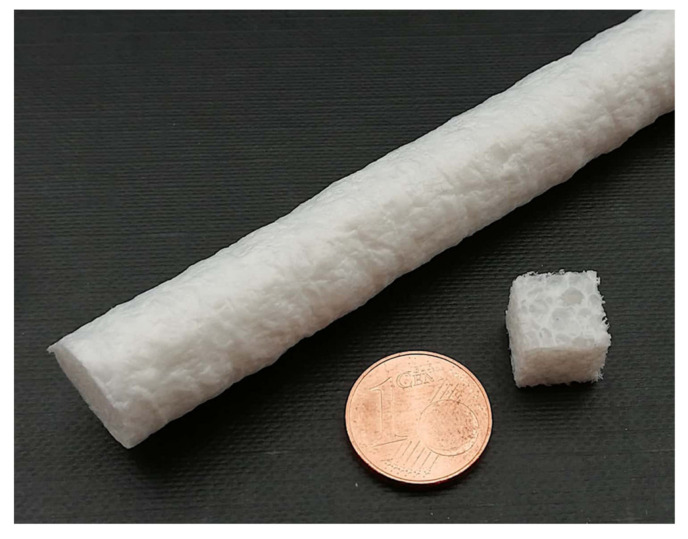
Image of the foam strand and the cubic foam sample with the length of 1 cm.

A universal test machine (Z050, ZwickRoell GmbH & Co. KG, Ulm, Germany) was used to determine the stress–strain curves and compressive strength (in agreement to ISO 844:2014) of cubic foam samples with the length of 10 mm. Pictures of the cubic foam samples, which were prepared from the foam strands are shown in Figure A5. Samples were exposed to a compressive load against to the extrusion direction and under a maximum strain of 30% allowing one to determine compressive strength values for each sample. Test speed was set as 1 mm/min. At the start of the test, 1 N preload was applied to guarantee a full contact between the foam samples and the plates of the machine. At least eight samples per material were tested to find the most representative stress–strain curve and to provide the average compressive strength value of corresponding PBT foams.

#### 2.4.4. Degree of Crystallinity

Differential scanning calorimetry from Mettler Toledo DSC/SDTA 821 e (Columbus, OH, USA) was conducted to determine the crystallinity of mPBT foams with regard to the effect of BTA type and concentration. The measurements were carried out under nitrogen atmosphere with a heating rate of 10 K/min from room temperature to 260 °C. The degree of crystallinity of foams was calculated using an enthalpy of fusion of 140 J/g for 100% crystalline PBT [60].

#### 2.4.5. Open Cell Content

A gas pycnometer (Ultrafoam 1000, UPY-15F, Quantochrome Instruments, Boynton Beach, FL, USA) was used to estimate the open-cell content of the foams according to ASTM D-2856. A cell with a volume of 10 cm^3^ was used for the measurement and the foam samples were exposed to 0.6 bar nitrogen. Three samples for each BTA type and concentration were measured and three runs were performed for each sample.

## 3. Results and Discussions

### 3.1. Influence of BTAs on Extensional Viscosity of mPBT

**Figure 3:**
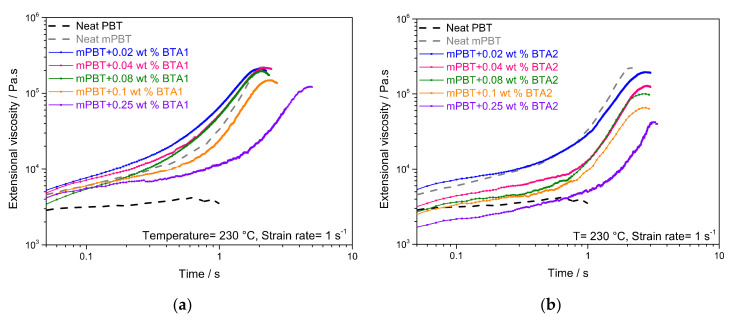
Extensional viscosity graphs of the neat PBT, neat modified PBT (mPBT) and mPBT with different concentrations of (**a**) BTA1 and (**b**) BTA2.

Prior to the foaming experiments, rheological parameters were determined, because extensional viscosity analysis of mPBT is crucial to correlate the strain hardening behavior with the foamability of mPBT and foam cell stabilization. The strain hardening coefficient (S) provides a quantitative estimation of the strain hardening behavior of polymers for a particular value of time and the Hencky strain. A higher S was associated with an enhanced melt strength offering an advantage when the polymer was exposed to elongation at high extensional strain rates as in the foam extrusion process. Improvement of the melt strength by achieving a pronounced strain hardening via chain extension of linear PBT with epoxy CE was already reported [6]. To evaluate the influence of the BTAs on the melt strength of mPBT, extensional viscosity curves of the neat PBT, neat mPBT and mPBT with various concentrations of BTA1 and BTA2 were measured at 230 °C and extensional strain rate of 1 s^−1^ and are shown in Figure 3.

Apart from neat PBT, all materials exhibited a rapid increase of the extensional viscosity with time, which was associated to strain hardening due to the chain extension and branching caused by the CE. This finding is in agreement with literature reports [6] and is an indication of an efficient chain modification of PBT. Up to a concentration of 0.1 wt % BTA1, a slight increase in extensional viscosity compared to the neat mPBT was seen (Figure 3a). At 0.1 wt % BTA1, a decrease in extensional viscosity and retarded strain hardening behavior started and got more pronounced at 0.25 wt % BTA1. This may be attributed to an incomplete solubility of the BTA in the PBT melt at a too high BTA concentration. These non-dissolved remaining aggregates may interfere with the branched chains causing a decreased extensional viscosity and retarded strain hardening behavior [61]. Figure 3b shows that compared to the neat mPBT, 0.02 wt % BTA2 features similar extensional viscosity but a noticeable retardation of the strain hardening closer to the time of 2 s, which is an approximate foam expansion time during foam extrusion [5]. mPBT with 0.04 wt % of BTA2 shows a decreased extensional viscosity compared to the neat mPBT. Further BTA2 addition up to 0.25 wt % leads to lower viscosities with more retarded strain hardening. This can be explained with the similar phenomenon as in the case of BTA1.

**Figure 4:**
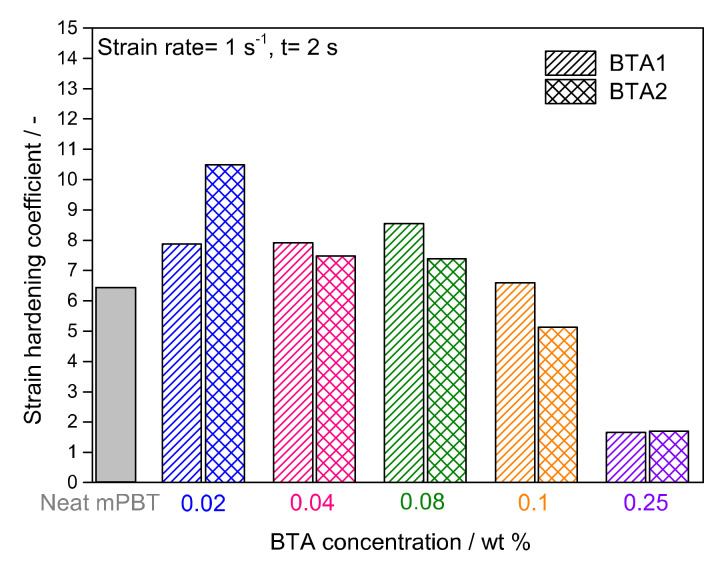
Strain hardening coefficients of mPBT and mPBT with BTA1 and BTA2 for a Hencky strain rate of 1 s^−1^ and a time of 2 s.

Furthermore, to determine whether BTAs provide additional enhancement in melt strength quantitatively, S values of each material were calculated according to Equation (1) for a Hencky strain rate of 1 s^−1^ and a time of 2 s. The calculated S values are shown in Figure 4 and demonstrated that the strain hardening behavior of mPBT was influenced by the BTAs.

In comparison with the neat mPBT, the highest increase of 32% and 64% in S were achieved by 0.08 wt % BTA1 and 0.02 wt % BTA2, respectively. While BTA1 concentrations from 0.02 to 0.08 wt % result in an enhanced melt strength, the S value started to decrease with 0.1 wt % and got much lower with 0.25 wt % of BTA1. A similar behavior can be seen for BTA2, where S reached its maximum at a concentration of 0.02 wt %. Addition of 0.04 and 0.08 wt % of BTA2 lead to deteriorated melt strength and a more pronounced decrease in S was observed for higher concentrations of BTA2. The difference in the maximal increase of S at the concentration of 0.02 wt % and 0.08 wt % for BTA2 and BTA1, respectively, was attributed to the different chemical structure, since BTAs based on 1,3,5-triaminobenezene (BTA1) featured better solubility in the PBT melt than BTAs based on trimesic acid (BTA2) [50]. Moreover, the fiber diameter of the supramolecular nanofibers also depends on the concentration and cooling rate [50]. Thus, we assume that under appropriate conditions, a percolated fiber network with submicron diameters is present in the polymer melt, which may lead to an enhancement in strain hardening behavior of mPBT.

### 3.2. Optimization Study of the Process Parameters

**Figure 5:**
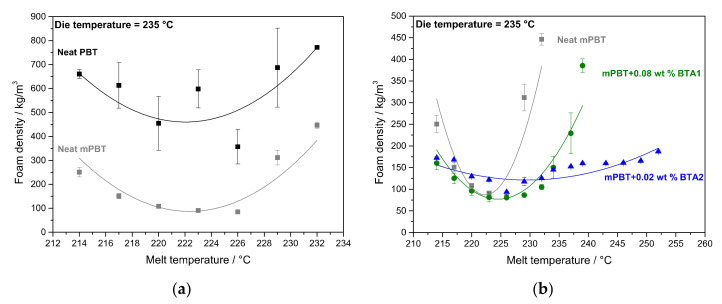
Change in foam density of (**a**) neat PBT and mPBT and (**b**) neat mPBT, mPBT + 0.08 wt % BTA1 and 0.02 wt % BTA2 by altering melt temperatures at the exit of the B-Extruder at constant die temperature of 235 °C. Samples used for foam density measurements were directly cut as slices from foam struts as extruded out of round-die (with skin layer) and the solid lines are guides to the eye.

To optimize the process parameters for foam extrusion, several foam strands based on neat mPBT and mPBT with selected concentrations of 0.08 wt % of BTA1 and 0.02 wt % of BTA2, and 2 wt % of s-CO_2_ were processed. Neat PBT was produced in the same manner as a reference material. Different processing temperature windows of the neat PBT and the mPBT without and without the two BTA concentrations were established by varying melt temperatures at the exit of the B-Extruder (Figure 5).

The neat PBT foam shows quite fluctuated density values with large deviations compared to the mPBT foams (Figure 5a). At 226 °C, the foam density of neat PBT reaches its minimum. Higher densities at temperatures lower than 226 °C may be the result of a faster solidification of the polymer melt restricting a proper expansion. At temperatures higher than 226 °C, increased foam densities might be due to low melt viscosity and strength causing fast diffusion of CO_2_ out of the extrudate and cell coalescence [62].

In contrast to the neat PBT foam, the modification of PBT with 0.6 wt % CE (mPBT) improves foamability with smaller deviations in density values, which can be associated to more uniform foam samples. Importantly, a great reduction in foam density induced by the modification of the PBT is noticeable. However, mPBT polymer foams with low foam density can be achieved only in a small processing window between 216 and 226 °C. mPBT with 0.02 wt % of BTA2 possess similar foam densities around 100 kg/m^3^ and mPBT with 0.08 wt % of BTA1 possesses also lower foam densities around 80 kg/m^3^ (Figure 5b). Noticeably, with 0.08 wt % of BTA1 a stable processing window between 220 and 232 °C and even with only 0.02 wt % of BTA2 between 220 and 260 °C was found. The addition of the BTAs clearly widened the processing temperature window of mPBT due to a control of extensional viscosity achieved by the presence of supramolecular nanofibers. Compared to BTA1, BTA2 provides an even more stable processing window considering a noticeable increase in foam density of mPBT with 0.08 wt % BTA1 at 234 °C, which may be attributed to non-assembled or disassembled nanofibers of BTA1 at these high temperatures.

This data show that the post-modification of the PBT reduced the foam density significantly, the foaming temperature was more crucial for achieving low-density foams, and the addition of the BTAs clearly contributed to the stability of the foaming process. We also identified for the mPBT foams with selected concentrations of BTA1 and BTA2 an optimum melt temperature range at around 226 °C.

### 3.3. Influence of BTAs on the Density and Morphology of Foams

**Figure 6:**
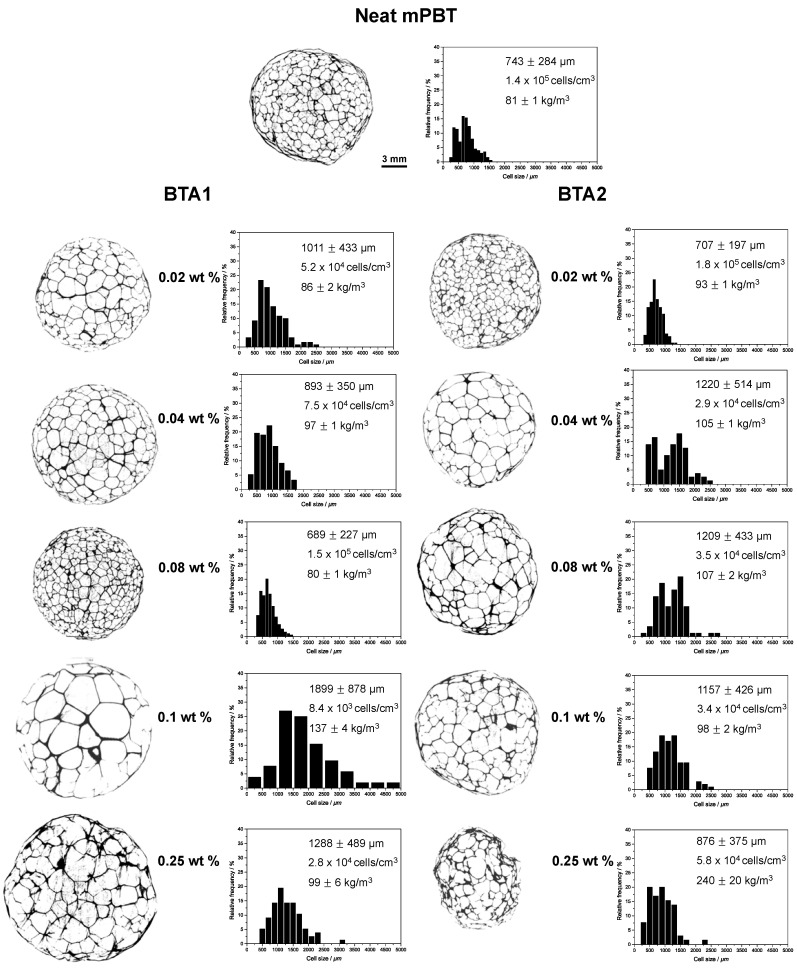
Overview µ-CT images of the neat mPBT foam and mPBT foams nucleated with different concentrations of BTA1 and BTA2 together with their corresponding cell size distributions, mean cell sizes, cell densities and foam densities. The same scale bar (3 mm) is shown for all of the images.

To evaluate the influence of the BTAs on the PBT foam morphology, we investigated foam specimens by means of µ-CT. PBT foams based on the neat reference PBT featured a too high foam density and a too undefined morphology preventing proper analysis after µ-CT imaging (Figure A5). An overview of the µ-CT images of the mPBT foam and mPBT foams prepared with 0.02, 0.04, 0.08, 0.1 and 0.25 wt % BTA1 and BTA2 with the corresponding cell size distributions are depicted in Figure 6. All of the samples were produced at the die temperature of 235 °C and melt temperature at around 226 °C.

For both BTA1 and BTA2, we found an optimum concentration providing the best morphology, which were 0.08 wt % and 0.02 wt %, respectively. In comparison with the neat mPBT, the addition of 0.02 and 0.04 wt % BTA1 resulted in larger mean cell sizes with broader deviation. mPBT with 0.08 wt % of BTA1 featured about a 7% decrease in cell size (689 ± 227 µm) compared to the mPBT (743 ± 284 µm) leading to the highest cell density of 1.5 × 10^5^ cells/cm^3^ by keeping the foam density constant. Although the decrease seems not to be very large, the cell size distribution gets narrower leading to a more uniform cell morphology. To the best of our knowledge, this is the lowest achieved foam density (80 kg/m^3^) for PBT foams with the smallest cell size and narrowest cell size distribution. Figure 6 show that a further increase in BTA1 concentration leads to non-uniform foams with larger cells and higher foam densities. One can expect that mPBT foams with 0.1 and 0.25 wt % BTA1 had lower foam density due to larger cells and expansion compared to the one with mPBT with 0.08 wt % BTA1. However, to have a stable process, the screw speed of the B-Extruder was adjusted to the material to avoid rapid increase in barrel pressure and sudden shutdown of the extruder. Therefore, at higher BTA 1 concentrations (0.1 and 0.25 wt %), the screw speed was increased to 7 rpm while it was set as 6 rpm for lower BTA1 concentrations and neat mPBT. Yet, increased screw speed led to greater shear forces, decreased residence time of the polymer in the extruder resulting in cell coalescence and foams with thicker cell walls and struts. mPBT foams with BTA2 follows a similar manner reaching the minimum cell size (707 ± 197 µm), highest cell density of 1.8 × 10^5^ cells/cm^3^ and the most uniform cell morphology at an optimum concentration of 0.02 wt %. Similarly, further increase in the BTA2 concentration caused coarser cellular structures and higher foam densities. Interestingly, the morphological results coincide with extensional viscosity fixture graphs (Figure 3) and resulting S coefficients (Figure 4) where mPBT with 0.08 wt % BTA1 and 0.02 wt % BTA2 featured the highest S coefficients. Thus, we assume that the increase in the extensional viscosity due to the presence of the proper concentration of nanofibers, improves the foamability and thus the foam morphology. When the best foams of mPBT with BTA1 and BTA2 were taken into consideration, both BTAs resulted in similar mean cell sizes. While 0.02 wt % BTA2 gave rise to more homogeneous foam morphology (lower standard deviation in mean cell size) when only using a quarter of the BTA1 concentration, the foams with 0.08 wt % BTA1 facilitated further density reductions up to 14%.

### 3.4. Influence of BTAs on Compression Strength and Other Properties of Foams

**Figure 7:**
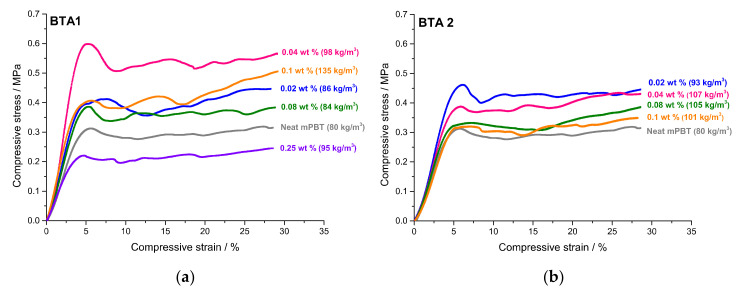
Compressive stress–strain curves of the neat mPBT foam and mPBT foams with various concentrations of BTA1 (**a**) and without 0.25 wt % BTA2 (**b**).

**Figure A6:**
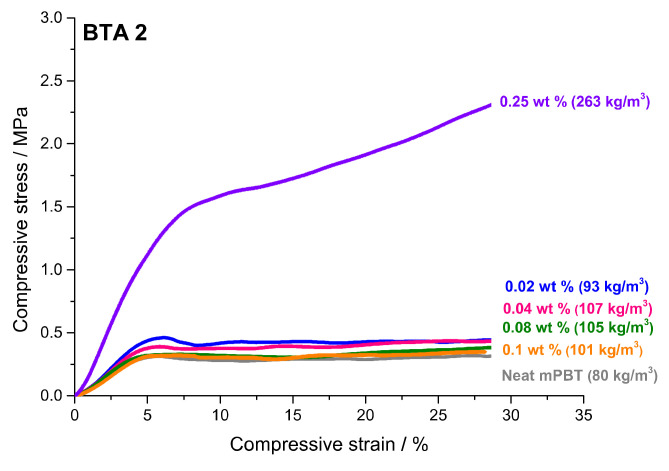
Compressive stress–strain curves of the mPBT foam and mPBT foams with all the concentrations of BTA2.

Besides foam density and morphology, compressive strength is also one of the most significant foam properties considering the applications (i.e., building and construction) of semicrystalline polymer foams. Therefore, the key is to achieve PBT foams with uniform foam morphology and low foam density without sacrificing compressive strength and stiffness. It is well known that the compressive strength is affected by foam density [63], foam morphology including cell size and cell size distribution [64], degree of crystallinity [65] and open-cell content [63]. In the case of foams nucleated by fiber like additives such as BTAs, the intrinsic reinforcing effect of the additive must be also considered. Figure 7 shows compressive stress–strain curves of the neat mPBT foam and mPBT foams with various concentrations of BTA1 and BTA2, respectively. The given densities refer to the density of each specimen showing the most representative stress–strain curve. Foams based on mPBT with 0.25 wt % of BTA2 were omitted and shown for completeness in Figure A6, because of the very large values of the compressive strength. This large deviation can be explained by its more than 3-times higher density rendering a comparison with the other foams difficult [63].

Figure 7 demonstrates that all other foam samples follow the typical behavior of elastomeric, closed-cell foams under compression forces. The initial linear elasticity appears at the low strains due to the elastic bending of cell walls and stretching of faces (membranes), followed by a plateau region. The stress where buckling of the struts and crushing of the cells (plastic collapse) starts is defined as the compressive strength. Obviously, compressive strength values of the foams are not directly proportional with the foam densities. This is reasonable considering other properties like morphology, degree of crystallinity and open cell content of the foams influencing the compressive properties. To make a more concise interpretation regarding the compressive strength and to subtract the contribution of the foam density, compressive strength values were normalized according to the technique described elsewhere [49,66]. For the sake of precision, the foams with the closest densities in the range of 80–110 kg/m^3^ were considered for the normalization study. The compressive strength values of both BTA1 and BTA2 were normalized at minimum, average and maximum foam density. It is noteworthy that two separate normalization studies were performed for mPBT foams with BTA1 and BTA2. Table 2 indicates the compressive strength values of all foams contributed to the normalization study and the normalized values at minimum, average and maximum density for BTA1 and BTA2.

**Figure 8:**
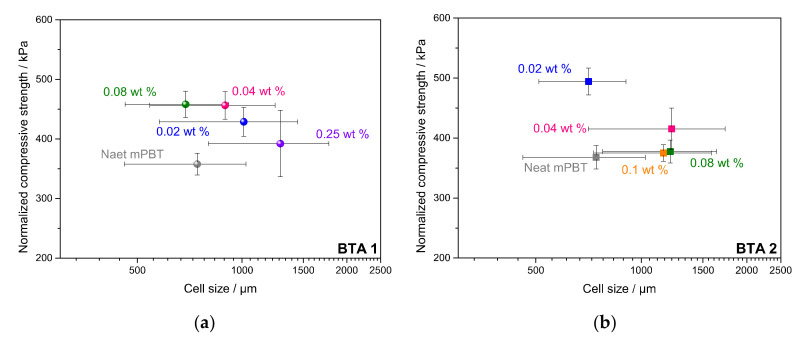
Influence of cell size on the normalized compressive strength at minimum density (80 kg/m^3^) of the neat mPBT foam and mPBT foams with (**a**) BTA1 and (**b**) BTA2. Normalization study was performed separately for BTA1 and BTA2 with various concentrations.

Table 2 shows that normalized compressive strength behaved in the same manner for both BTA1 and BTA2 regardless of which density (minimum, average or maximum) was considered for the normalization study. Differently, the values that were normalized at a minimum foam density of 80 kg/m^3^ had smaller standard deviations and thus show higher precision and reliability compared to those that were normalized at average and maximum densities. There is one BTA concentration for each BTA1 (0.08 wt %) and BTA2 (0.02 wt %) leading to the highest normalized compressive strength values. According to the normalized compressive strength values at 80 kg/m^3^, 0.08 wt % BTA1 and 0.02 wt % BTA2 featured 28% and 34% improvement in normalized compressive strength compared to neat mPBT. Similar to the foam with 0.08 wt % BTA1, a 27.5% increase in the normalized strength was achieved with 0.04 wt % BTA1. These findings were in a very good agreement with the previously mentioned results. As expected, the BTA concentrations leading to the best foamability (highest S) and finest morphology resulted in foams with enhanced compressive properties. Nevertheless, to make more concrete interpretations of whether the improvement in compressive strength is induced by the intrinsic reinforcing effect of BTA fibers, other parameters like the cell size (Figure 8), degree of crystallinity and open-cell content (Table 3) of the foams have to be analyzed. Figure 8 depicts the influence of the cell size of the neat mPBT foam and mPBT foams with 0.02, 0.04, 0.08 and 0.25 wt % BTA1 and 0.02, 0.04, 0.08 and 0.1 wt % BTA2 having normalized compressive strength at 80 kg/m^3^.

**Figure 9:**
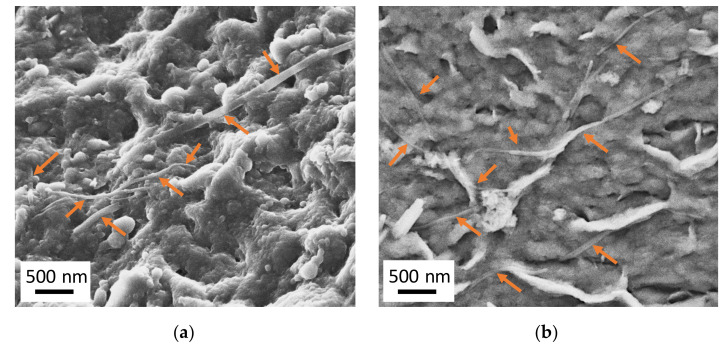
FE-SEM micrographs of the hydrolyzed mPBT foam with (**a**) 0.08 wt % BTA1 and (**b**) 0.02 wt % BTA2.

As seen in Table 2 and Figure 8a, addition of 0.02 wt % BTA1 already improved the compressive strength of mPBT and a further increase of BTA1 to 0.04 wt % and 0.08 wt % resulted in the highest normalized strength values. Surprisingly, despite its greater mean cell size, mPBT foam with 0.04 wt % BTA1 resulted in significantly higher normalized compressive strength compared to the one for the neat mPBT. Apparently, an increase in BTA1 concentration from 0.04 to 0.08 wt % could not increase the normalized compressive strength of foams. However, slightly lower standard deviations in compressive values due to the improved morphology (smaller cell sizes and narrower cell size distribution) were noticeable (Table 2 and Figure 6). When the foams of mPBT and mPBT + 0.08 wt % BTA1 were compared, it could be clearly seen that although both had a very similar mean cell size and cell size range, mPBT foam with 0.08 wt % BTA1 depicted significantly higher normalized strength. The highest BTA1 concentration of 0.25 wt % caused deteriorated compressive strength, which might be due to the non-uniform foam morphology resulting in the largest standard deviations in normalized compressive strength values. This might be explained by insoluble BTA1 aggregates due to limited solubility in the mPBT melt. Furthermore, mPBT foam with 0.02 wt % BTA2 possessed noticeably higher normalized compressive strength compared to the neat one (Figure 9b) while both show a similar mean cell size and cell size range. Further increase in BTA2 concentration up to 0.1 wt % BTA2 caused reduced normalized compressive strength values, which was in agreement with the findings from extensional viscosity and foam morphology analyses. All in all, cell size and cell morphology of the foams had a significant effect on compressive properties. However, considering that the mPBT foam and foams with 0.08 wt % BTA1 and 0.02 wt % BTA2 had a very similar mean cell size and cell size range, we attributed that the improvement in compressive properties might be due to an intrinsic reinforcing effect of BTA fibers, a higher degree of crystallinity or a lower open-cell content of all the foams. To be able to distinguish the effecting parameters easily, the values of the degree of crystallinity and open-cell content of the mPBT foam and foams with 0.08 wt % BTA1 and 0.02 wt % BTA2 are tabulated in Table 3.

Table 3 depicts that the degree of crystallinity was not affected by both BTA1 and BTA2 and crystallinity was dominated by the high strain forces applied during the foam extrusion process. Similar behavior was shown already by Mörl et al. [3] where they reported that addition of BTA in i-PP does not change the crystallinity of the foams. When the open-cell content values of the foams were seen, 0.08 wt % BTA1 led to almost the same open-cell content of around 42% as for mPBT. Nevertheless, 0.02 wt % BTA2 reduced the open-cell content of mPBT from around 42%–30%. Therefore, when all the effects (foam density, cell size and cell size distribution, degree of crystallinity and open-cell content) were taken into account, we attributed that the enhanced compressive strength of mPBT foams with 0.08 wt % BTA1 was achieved by the intrinsic reinforcing effect of BTA1 fibers, which were located on the cell walls (Figure 9) and struts providing increased buckling resistance by retarding the face stretching and edge bending. The nanofibers contributed to the stress transfer through the foam, which led to increased compressive strength. Differently, improvement in compressive strength with 0.02 wt % BTA2 might be explained by a lower content of open-cells compared to mPBT. Besides that, we believe that BTA2 also contributes to an increase in compressive strength by reinforcing the foam, due to its fiber-like structure. Aside from PBT, the intrinsic reinforcing effect of BTA fibers has already been declared for PS [49] and i-PP [3].

As shown in Figure 9, the percolated-network structures of BTA fibers are embedded within the cell walls of the mPBT foams. The BTA1 fibers have relatively larger diameters of around 75–85 nm compared to those of BTA2 fibers (around 45–55 nm), which might be due to higher BTA1 concentration and different solubility in the mPBT melt induced by the different chemical nature of the BTAs.

## 4. Conclusions

In this study, for the first time, we established a sustainable route to obtain low-density, extruded mPBT foams using supramolecular nanofibers based on BTA1 and BTA2 featuring enhanced compressive properties. The influence of the BTA type and their concentrations on strain hardening behavior, foamability and foam morphology was determined. Compared to the mPBT foam, 0.08 wt % BTA1 resulted in 32% greater S while 0.02 wt % BTA2 led to 64% increase in S. The improved strain hardening behavior with BTAs featured superior foamability together with a significant improved process stability resulting in more uniform foam morphologies with relatively small cells. For each BTA type, one concentration was found to be optimum, leading to the smallest cell size and narrowest cell size distribution and lowest foam density: 0.08 wt % for BTA1 and 0.02 wt % for BTA2. The compressive strength of the mPBT foams with various concentrations of BTA was normalized at the lowest, average and maximum foam densities. According to the normalized compressive strength values at 80 kg/m^3^, the foams with the best morphologies provided mPBT foams with enhanced compressive properties. In comparison with the normalized compressive strength of mPBT, 0.08 wt % BTA1 and 0.02 wt % BTA2 resulted in a 28% and 15% increase in normalized compressive strength, respectively. To gain a deeper understanding of the improved compressive properties, the degree of crystallinity and open-cell content of the foams were also analyzed. A thermal analysis of the foams showed that the degree of crystallinity did not depend on the BTA type and concentration and was only affected by the strain forces occurring during the process and featured almost the same values of around 30%. According to the open-cell content results, mPBT and mPBT foams with 0.08 wt % BTA1 exhibited very close open-cell contents of around 42% while 0.02 wt % BTA2 led to foams with increased close-cell content of around 70%. Therefore, we attributed that the improvement in compressive properties of the foam with 0.08 wt % BTA1 was due to the intrinsic reinforcing effect of BTA2 fibers located in the cell walls, considering that the other foam properties were very similar to those of mPBT foam. Furthermore, enhancement of compressive properties of mPBT foam with 0.02 wt % BTA2 can be explained by higher close-cell content. Aside to the lower-open cell content, we assumed that there might be an additional intrinsic reinforcing effect of BTA2 fibers on improved compressive strength as in the case of BTA1. Considering all factors, particularly compression performance at the low-foam density, 0.08 wt % of BTA1 was found to be the most optimum material, because it provides a significant improvement in compressive strength while featuring the lowest foam density among all materials.

## Figures and Tables

**Table 1:** Processing parameters for the reactive foam extrusion process.

Melt Temperature [°C]	Die Temperature [°C]	Screw Speed [rpm]	Throughput [kg/h]	s-CO_2_ Content [wt %]
226	235	6–7	4	2

**Table 2:** Real compressive strength values and the normalized values of the neat mPBT foam and mPBT foams with BTA1 and BTA2 at minimum, average and maximum foam densities.

**Sample**	**Compressive Strength (CS) [kPa]**	**Normalized CS at Minimum Density of 80 kg/m^3^ [kPa]**	**Normalized CS at Average Density of 88 kg/m^3^ [kPa]**	**Normalized CS at Maximum Density of 99 kg/m^3^ [kPa]**
Neat mPBT	368.1 ± 21.2	357.8 ± 18.4	428.1 ± 22.1	534.1 ± 27.5
0.02 wt % BTA1	486.2 ± 22.4	428.8 ± 24.3	512.9 ± 29.1	640.2 ± 36.2
0.04 wt % BTA1	650.2 ± 33.2	456.3 ± 23.3	545.9 ± 27.8	681.2 ± 34.7
0.08 wt % BTA1	460.4 ± 15.5	458.1 ± 22.1	547.9 ± 26.4	683.8 ± 33.1
0.25 wt % BTA1	584.9 ± 54.4	392.2 ± 55.9	469.2 ± 66.8	585.4 ± 83.4
**Sample**	**Compressive Strength (CS) [kPa]**	**Normalized CS at Minimum Density of 80 kg/m^3^ [kPa]**	**Normalized CS at Average Density of 97 kg/m^3^ [kPa]**	**Normalized CS at Maximum Density of 107 kg/m^3^ [kPa]**
Neat mPBT	368.1 ± 21.2	367.9 ± 21.2	371.5 ± 21.3	373.3 ± 21.5
0.02 wt % BTA2	497.8 ± 22.6	494.2 ± 22.4	499.1 ± 22.6	501.5 ± 22.7
0.04 wt % BTA2	421.1 ± 35.4	415.3 ± 34.9	419.4 ± 35.2	421.4 ± 35.4
0.08 wt % BTA2	383.1 ± 19.5	377.6 ± 19.1	381.3 ± 19.2	383.2 ± 19.4
0.1 wt % BTA2	381.8 ± 12.4	375.1 ± 13.9	378.7 ± 14.1	380.5 ± 14.1

**Table 3:** Degree of crystallinity and open-cell content of the neat mPBT foam and foams with 0.08 wt % BTA1 and 0.02 wt % BTA2.

Sample	Degree of Crystallinity [%]	Open Cell Content [%]
Neat mPBT	32.4 ± 0.3	42.2 ± 0.8
0.08 wt % BTA1	28.8 ± 1.4	42.3 ± 0.5
0.02 wt % BTA2	30.1 ± 1.1	29.9 ± 1.1
